# Partial Horner’s Syndrome as a Manifestation of Carotid Artery Dissection: A Case Report and Diagnostic Approach

**DOI:** 10.7759/cureus.86775

**Published:** 2025-06-26

**Authors:** Ikram El Farsi, Vinod Warrier, Veena Muraleedharan

**Affiliations:** 1 Internal Medicine, Mid and South Essex NHS Foundation Trust, Southend-on-Sea, GBR; 2 Ophthalmology, Mid and South Essex NHS Foundation Trust, Southend-on-Sea, GBR

**Keywords:** ct (computed tomography) imaging, general neurology, headache, horners syndrome, non-traumatic carotid artery dissection

## Abstract

Horner's syndrome is a neurological condition resulting from a disruption in the sympathetic pathways supplying the head and neck. This syndrome often occurs secondary to various aetiologies, including trauma, neoplastic compression, or vascular pathology such as carotid artery dissection. Although the clinical presentation is well-defined, Horner’s syndrome remains relatively rare, and there are limited established management guidelines. Of particular concern is the acute onset of occipital pain in association with partial Horner's syndrome, as it raises the suspicion of cervical artery dissection, a potentially life-threatening condition. Prompt diagnosis using MRI angiography or CT angiography of the neck is critical, as routine CT head imaging may not be sufficient to identify the underlying pathology. We present a case of a 44-year-old female who presented with left-sided occipital headache, neck pain, and anisocoria, which led us to believe this was a manifestation of Horner's syndrome. The patient was treated with dual antiplatelet therapy, resulting in complete resolution of the dissection on follow-up imaging. This case underscores the importance of considering carotid artery dissection (CAD) in the differential diagnosis of Horner’s syndrome, particularly in the absence of trauma. It highlights the need for urgent neuroimaging and a multidisciplinary approach to diagnosis and treatment to improve patient outcomes and prevent life-threatening sequelae.

## Introduction

Horner’s syndrome is a group of symptoms resulting from disruption along the sympathetic nervous system, which innervates regions like the head, eyes, and neck areas [1,2]*.* This disruption typically presents with ipsilateral miosis, ptosis, and facial anhidrosis on the affected side. These symptoms occur due to unopposed parasympathetic activity resulting from the loss of sympathetic tone, leading to the dominance of the iris constrictor muscle, causing miosis [3]. Ptosis results from the impact on the superior tarsal muscle, responsible for a minor degree of upper eyelid elevation, while anhidrosis results from sympathetic denervation of sweat glands, which is less common in postganglionic cases [3,4].

The oculosympathetic pathway can be anatomically divided into three segments: central, preganglionic, and postganglionic. First-order neurons start in the hypothalamus, travel through the brainstem, and terminate at the ciliospinal centre of Budge, located in spinal segments C8 to T2. Second-order neurons exit the spinal cord, cross over the lung apex, and synapse at the superior cervical ganglion. Third-order neurons then travel along the internal carotid artery to the ophthalmic division of the trigeminal nerve, ultimately innervating the iris dilator and Müller’s muscles. A disruption at any point in this pathway can result in Horner’s syndrome, with clinical signs varying based on the lesion's location.

The aetiology of Horner’s syndrome includes a spectrum of benign to serious conditions, such as infections, malignancies, and vascular diseases like carotid or vertebral artery dissection. Timely identification and localisation of the lesion are critical for effective management [5,6].

## Case presentation

A 44-year-old female presented to the emergency department with a 5-day history of new-onset left-sided occipital headache and neck pain. The headache was described as initially sharp and severe, rated at 8/10, gradually decreasing to a dull ache rated at 3/10 intensity on a pain scale. Associated symptoms included anisocoria, which the patient noticed upon self-examination, and difficulty focusing her left eye in the evening despite maintaining a Snellen visual acuity of 6/6 bilaterally. She denied any history of trauma, recent infection, or systemic illness. She had no significant past medical history.

Neurological examination revealed partial ptosis and miosis (pupil size 3 mm) on the left side with no detected abnormality on the right side (pupil size 4 mm), with no evidence of facial anhidrosis, indicating a partial Horner’s syndrome. A dark room test showed delayed dilation of the left pupil compared to the right, further supporting the diagnosis. Due to the acute onset of headache and neck pain with associated partial ptosis and miosis, a cervical artery dissection was strongly suspected as a primary differential due to its known association with the above symptoms.

**Figure 1:**
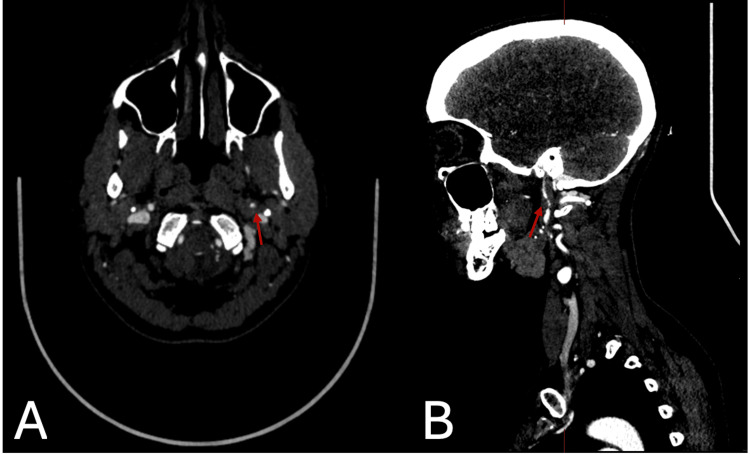
Axial (A) and sagittal (B) CT imaging demonstrating circumferential narrowing of the left internal carotid artery (ICA), with increased density in the vessel wall consistent with an intramural haematoma.

**Figure 2:**
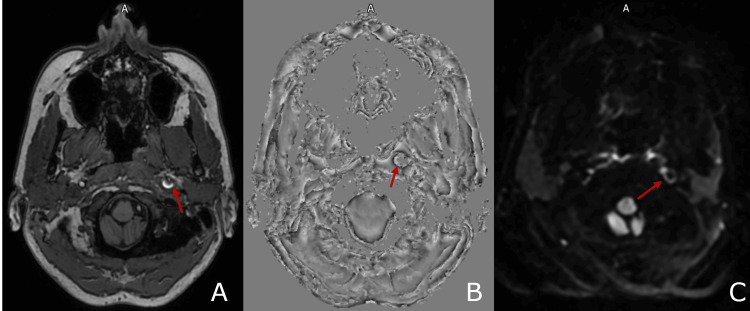
T1 (A), susceptibility-weighted image (B), and diffusion-weighted sequences (C) indicating left ICA intramural haematoma T1, susceptibility-weighted image, and diffusion-weighted sequences reveal signal return from the left internal carotid artery (ICA) lumen - a classic crescent sign is evident, which is indicative of dissection caused by intramural hematoma

CT imaging, shown in Figure 1, revealed circumferential narrowing of the left internal carotid artery (ICA) with increased density, consistent with an intramural haematoma. The patient's MRI imaging, shown in Figure 2, demonstrated that the left internal carotid artery in its distal cervical segment was enlarged and showed a heterogeneous signal with alteration of the normal flow void. To confirm the diagnosis, a CT angiogram of the aortic arch and neck was performed, revealing a dissection of the left internal carotid artery, located 2 cm from the bulb and extending to the carotid canal. The dissection resulted in a 90% luminal narrowing, though distal perfusion was preserved. Imaging with a CT head and MRI brain ruled out additional intracranial pathology, such as ischaemic stroke or aneurysm.

**Figure 3:**
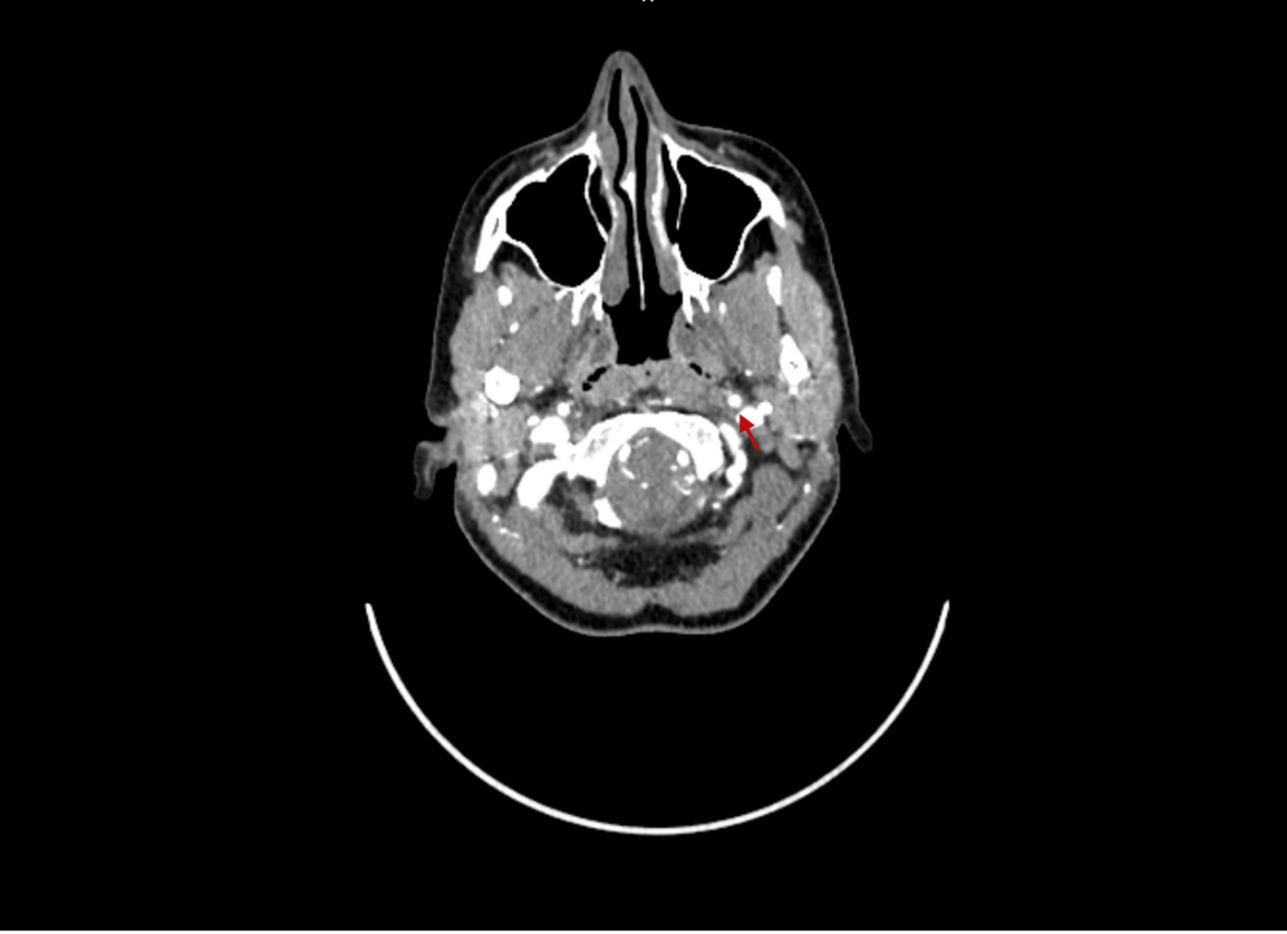
Follow-up CT angiography after four months Follow-up CT angiography after four months demonstrated the resolution of the previously noted circumferential narrowing and mural thickening of the left internal carotid artery (ICA), consistent with resolved intramural haematoma. The lumen is now of normal calibre with no residual thrombus or contrast flow limitation.

A multidisciplinary team comprising vascular surgeons, neurologists, and stroke specialists was engaged in managing the patient. Due to the confirmed diagnosis of carotid artery dissection and the associated Horner’s syndrome, a decision was made to initiate dual antiplatelet therapy with aspirin and clopidogrel. This therapeutic approach aimed to reduce the risk of thromboembolic complications and stabilise the dissection. Follow-up imaging was scheduled at four months to assess the resolution or progression of the dissection and to evaluate the effectiveness of the therapeutic regimen. The follow-up imaging in Figure 3 revealed a complete resolution of the previously noted dissection.

## Discussion

In this case report, the presenting complaint of neck pain and headache could have been dismissed as benign symptoms. However, on further examination, the symptoms masked a more sinister pathology. When viewed in isolation without other preceding signs, the symptoms raised strong clinical suspicion of neurological dysfunction. In a study of 161 symptomatic acute Horner's syndrome patients, neck pain was noted in 26% of those with internal carotid artery dissection (ICAD) and 46% of those with vertebral artery dissection (VAD), frequently manifesting as posterior neck pain in VAD cases and anterolateral neck pain in ICAD cases. This symptom, particularly when accompanied by headaches, which were present in 68% of ICAD patients and 69% of VAD patients, can easily be misattributed to common musculoskeletal issues or migraine-related phenomena [7].

In contrast, head or neck pain is a less frequent presenting symptom in Horner's syndrome. When it does occur, it is often misattributed to other pathologies, making it a relatively uncommon feature in clinical practice. In this case, the patient delayed seeking medical evaluation, mistakenly perceiving the reduced headache intensity as an indication of improvement. Her transition from a sharp, severe headache to a dull ache rated at 3/10 might have given her a false sense of security. This highlights the role, as clinicians, to recognise that a reduction in pain does not always correlate with a benign underlying condition. In severe cases precipitated by a vascular issue, the initial severity of the headache can sometimes diminish while serious pathology persists or evolves. A nuanced understanding of the significance of headache patterns, particularly associated with other neurological symptoms, is required to prevent missed diagnoses and improve patient outcomes [7,8].

Diagnostic imaging, particularly MRI angiography or CT angiography, plays a pivotal role in evaluating suspected cervical artery dissection. Within district general hospitals, digital subtraction angiography (DSA) is used less frequently due to the availability of non-invasive imaging techniques like CT and MR angiography. However, clinicians should be aware of DSA for detailed vascular assessment and intervention, especially when complex cases require specialist input or transfer to tertiary centres. Routine CT head imaging may fail to identify subtle or distal vascular abnormalities, highlighting the need for targeted imaging of the cervical vasculature [8]. In this case, the utilisation of CT angiography was instrumental in detecting the severe narrowing of the lumen in the left carotid artery and initiating timely management. The absence of facial anhidrosis in this patient’s presentation suggests that the lesion occurred distal to the superior cervical ganglion, sparing the sympathetic fibres responsible for innervating the sweat glands. This clinical clue was essential for narrowing the differential diagnosis and guiding the choice of imaging studies.

Pharmacological testing can assist in identifying the location of the lesion within the sympathetic pathway. Topical cocaine testing, which inhibits norepinephrine reuptake, typically reveals less dilation in the affected eye, confirming the presence of Horner’s syndrome. However, this tool is less frequently utilised due to issues with cost and regulation [9]. Upon confirmation of Horner’s syndrome, hydroxyamphetamine testing can differentiate between preganglionic and postganglionic lesions by stimulating norepinephrine release in the third-order neuron. Although historically once the standard, cocaine and hydroxyamphetamine tests have become less common due to availability issues and high costs [9,10].

The topical apraclonidine test has emerged as the most practical and accessible option, replacing these tests. The alpha-adrenergic agonist, which causes mydriasis in the affected eye due to denervation hypersensitivity, is widely used, though it may elicit false negatives in the early stages of Horner’s syndrome [9].

A collaborative approach involving neurology, radiology, vascular surgery, and ophthalmology ensures comprehensive assessment, precise diagnosis, and effective management strategies, which are essential in cases of suspected vascular dissection. Neurologists provide expertise in assessing neurological symptoms and are often the first to initiate advanced imaging, such as CTA or MRI, critical for identifying arterial dissection.

This patient was managed with dual antiplatelet therapy (aspirin and clopidogrel) to reduce the risk of thromboembolism and promote vessel healing, consistent with evidence from the Cervical Artery Dissection in Stroke Study (CADISS) trial. Radiologists then play a crucial role in interpreting these imaging results, focusing on subtle signs that might indicate a vascular abnormality or dissection, essential for timely intervention [11,12,13,14]. Should a vascular dissection be confirmed, vascular surgeons contribute significantly by evaluating the need for surgical versus conservative management.

Surgical or endovascular intervention, such as carotid stenting, is reserved for cases with persistent or worsening neurological symptoms, significant stenosis causing cerebral hypoperfusion, or failure of medical management [15]. Thus, the multidisciplinary team (MDT) framework fosters a cohesive treatment plan, allowing for adjustments based on symptom progression, ultimately enhancing diagnostic accuracy and optimising outcomes through a team-based, patient-centred approach.

## Conclusions

Horner’s syndrome requires a thorough and systematic evaluation to identify potentially serious underlying causes, such as carotid artery dissection. This case underscores the critical role of early recognition, as delayed diagnosis can lead to severe complications, such as ischemic stroke, leaving the patient with debilitating consequences. Patients presenting with acute headache and partial Horner’s syndrome should prompt a high degree of suspicion from clinicians for vascular pathology, necessitating urgent neuroimaging with CT or MR angiography.

This case emphasises the importance of considering carotid artery dissection (CAD) in patients with headache, neck pain, and Horner’s syndrome, even in the absence of trauma. Prompt recognition and imaging evaluation are crucial for early diagnosis and intervention. Multidisciplinary collaboration between acute medicine, vascular surgery, neurology, and radiology is essential for timely diagnosis and management, ultimately improving patient outcomes. Our patient’s successful outcome with dual antiplatelet therapy shows the effectiveness of conservative medical treatment in stabilising CAD, with follow-up imaging demonstrating complete resolution. This report highlights the need for increased clinical awareness to ensure prompt identification and management of Horner’s syndrome, preventing life-threatening sequelae.
